# Infections Caused by *Acinetobacter baumannii* in Recipients of Hematopoietic Stem Cell Transplantation

**DOI:** 10.3389/fonc.2014.00186

**Published:** 2014-07-14

**Authors:** Khalid Ahmed Al-Anazi, Asma M. Al-Jasser

**Affiliations:** ^1^Section of Adult Hematology and Oncology, Department of Medicine, College of Medicine and King Khalid University Hospital, King Saud University, Riyadh, Saudi Arabia; ^2^Central Regional Laboratory, Ministry of Health, Riyadh, Saudi Arabia

**Keywords:** *Acinetobacter baumannii*, hematological malignancy, hematopoietic stem cell transplantation, virulence, drug resistance

## Abstract

*Acinetobacter baumannii* (*A. baumannii*) is a Gram-negative, strictly aerobic, non-fermentative coccobacillus, which is widely distributed in nature. Recently, it has emerged as a major cause of health care-associated infections (HCAIs) in addition to its capacity to cause community-acquired infections. Risk factors for *A. baumannii* infections and bacteremia in recipients of hematopoietic stem cell transplantation include: severe underlying illness such as hematological malignancy, prolonged use of broad-spectrum antibiotics, invasive instrumentation such as central venous catheters or endotracheal intubation, colonization of respiratory, gastrointestinal, or urinary tracts in addition to severe immunosuppression caused by using corticosteroids for treating graft versus host disease. The organism causes a wide spectrum of clinical manifestations, but serious complications such as bacteremia, septic shock, ventilator-associated pneumonia, extensive soft tissue necrosis, and rapidly progressive systemic infections that ultimately lead to multi-organ failure and death are prone to occur in severely immunocompromised hosts. The organism is usually resistant to many antimicrobials including penicillins, cephalosporins, trimethoprim–sulfamethoxazole, almost all fluoroquinolones, and most of the aminoglycosides. The recently increasing resistance to carbapenems, colistin, and polymyxins is alarming. Additionally, there are geographic variations in the resistance patterns and several globally and regionally resistant strains have already been described. Successful management of *A. baumannii* infections depends upon appropriate utilization of antibiotics and strict application of preventive and infection control measures. In uncomplicated infections, the use of a single active beta-lactam may be justified, while definitive treatment of complicated infections in critically ill individuals may require drug combinations such as colistin and rifampicin or colistin and carbapenem. Mortality rates in patients having bacteremia or septic shock may reach 70%. Good prognosis is associated with presence of local infection, absence of multidrug resistant strain, and presence of uncomplicated infection while poor outcome is associated with severe underlying medical illness, bacteremia, septic shock, multi-organ failure, HCAIs, admission to intensive care facilities for higher levels of care, and culture of certain aggressive genotypes of *A. baumannii*.

## Overview of *Acinetobacter baumannii* Infections

### Introduction

*Acinetobacter baumannii* (*A. baumannii*) is a Gram-negative, strictly aerobic, non-fastidious, non-motile, catalase-positive, oxidase-negative, and non-fermentative coccobacillus. It is widely distributed in nature and environmental sources include: soil, water, vegetables, animals, and insects (1–3). The genus *Acinetobacter* comprises more than 30 different species. The four most common pathogenic types in humans are: *A. baumannii, A. calcoaceticus, Acinetobacter* genomic species 3, and *Acinetobacter* genomic species 13TU. These four species are very closely related and are difficult to be distinguished from each other by phenotypic properties (1–3). In 1911, Willem Beijerinck isolated an organism named *Micrococcus calcoaceticus* from soil after enrichment in a calcium-acetate-containing medium. The genus designation was initially proposed by Brisou and Prevot in 1954 then by Bauman et al in 1968. In the year 1974, the genus was finally listed in Bergey’s manual of clinical bacteriology and a single species, *A. calcoaceticus*, was described (1, 2).

### Sources of infections and virulence

Sources of *A. baumannii* infections include: skin and mucous membranes, burns and wounds, intravascular and urinary catheters, as well as gastrointestinal, urinary, and respiratory tracts. However, at times no source of infection or bacteremia can be identified (1, 3). Hospital sources of infection include: sinks, tables, mattresses, pillows, shower units, infusion pumps in addition to suction and resuscitation equipment (4).

The bacterium harbors a number of effective virulence factors that include: (1) attachment to and persistence on solid and dry surfaces, (2) ability to obtain nutrients such as iron, (3) adhesion and subsequent destruction of epithelial cells, (4) ability of some strains to produce gelatinases and proteinases that damage host tissues, (5) ability to colonize the skin of patients as well as health individuals without causing illness, and (6) ability to form biofilms that play an important role in the process of colonization (2).

### Risk factors for *A. baumannii* infections

*Acinetobacter baumannii* causes colonization, various infectious complications, and even epidemics. Community-acquired infections are less common than health care-associated infections (HCAIs) (2–4). There are several risk factors for *A. baumannii* infections and these are included in Table 1 (2–4).

**Table 1:** **Risk factors for *A. baumannii* infections**.

(1) Severe underlying illness, particularly hematological malignancy
(2) Critically ill patients admitted to ICU having endotracheal intubation and high APACHE score
(3) Prolonged antimicrobial therapy with carbapenems, fluoroquinolones, aminoglycosides, and third generation cephalosporins
(4) Infection or colonization of respiratory, urinary, and gastrointestinal tracts
(5) Burns and surgical wounds
(6) Diabetes mellitus
(7) Chronic lung disease
(8) Blood product transfusions
(9) Enteral feeding and contaminated parenteral solutions
(10) Circumstances of hospitalization: length of stay, high work load, and admission to wards with high density of infected or colonized patients
(11) Prematurity

*ICU, intensive care unit; APACHE, acute physiology and chronic health evaluation*.

### Clinical aspects of *A. baumannii* infections

The clinical manifestations of *A. baumannii* infections are very variable and include: non-specific features; soft tissue, skin, and wound infections; urinary tract infections; gastrointestinal tract (GIT) infections; respiratory tract infections including community-acquired and hospital-acquired or ventilator-associated pneumonia; infection of urinary or central venous catheters (CVCs); eye infections including keratitis and endophthalmitis; osteomyelitis; meningitis; endocarditis; and primary bacteremia where no source of infection is found (1–3, 5, 6). Infections caused by *A. baumannii* can be complicated by: extensive soft tissue necrosis, bloodstream infections, septic shock, acute respiratory distress syndrome (ARDS), disseminated intravascular coagulation (DIC), systemic or disseminated infection, multi-organ failure, and death (1, 3).

### Management of *A. baumannii* infections

A variety of tools are used in the diagnosis of *A. baumannii* infections. Swabs, septic screens, and surveillance cultures should be taken from various sites. Blood cultures should be taken peripherally and centrally in patients having indwelling intravascular catheters. Susceptibility studies and minimal inhibitory concentrations (MICs) should be performed on positive cultures. Molecular methods such as polymerase chain reaction (PCR) are very productive diagnostically. Radiological tools such as chest x-rays and computed tomography (CT) scans of chest, abdomen, and pelvis are helpful in determining the site of infection (5). A recent systematic review and meta-analysis on the impact of antibiotic MIC on infection outcome in patients having susceptible Gram-negative bacilli (GNB) revealed that there is an association between high MIC values within the currently accepted susceptible range and adverse outcome of infections caused by Gram-negative organisms. Therefore, not only susceptibility data but also MIC values are required to efficiently control infections caused by GNB (7).

Treatment of *A. baumannii* infections includes the following: (1) removal of vascular and urinary catheters, (2) surgical treatment such as drainage of abscesses and debridement of wounds, (3) use of appropriate antimicrobial agents, (4) growth factors in neutropenic patients, (5) ICU admission and mechanical ventilation may be required, and (6) transfusion of blood products in cytopenic patients (3). The drug of choice for treatment of *A. baumannii* infections is not yet established, so prospective and randomized controlled are needed (1, 3, 6, 8, 9). The most effective drugs in treating *A. baumannii* infections are: carbapenems such as imipenem and meropenem, β-lactam inhibitors such as ampicillin–sulbactam in addition to piperacillin–tazobactam and cephalosporins such as ceftazidime (1, 3, 6, 8, 9). For community-acquired *A. baumannii* (CAAB) infections, the following antibiotics have been reported to be effective: cephalosporins such as ceftazidime, cefepime and cefpirome; carbapenems; aminoglycosides; and fluoroquinolones (3).

New treatment options for *A. baumannii* infections include: (1) polypeptide antibiotics such as colistin, polymyxin B, and polymyxin E, (2) minocycline derivatives such as tigecycline, (3) new carbapenems such as doripenem, and (4) new generation cephalosporins such as ceftobiprole and ceftaroline (1, 3, 6, 8, 9). New experimental trials are needed to evaluate the activity and safety of peptides and other novel antimicrobial agents for *A. baumannii* infections (1, 3, 6, 8, 9). Treatment of complicated infections in severely ill and septic patients having *A. baumannii* is usually in the form of combination therapies that include: piperacillin–tazobactam and amikacin, piperacillin–tazobactam and colistin, or colistin and rifampicin (10).

Tigecycline is a first-in-class extended-broad-spectrum glycylcycline that has activity against many Gram-positive and Gram-negative organisms (11, 12). It overcomes the two key tetracycline resistance mechanisms, efflux pump, and ribosomal protection, and is unaffected by other bacterial mechanisms of resistance such as extended-spectrum β-lactamases (ESBL). Unfortunately, the use of tigecycline monotherapy has been associated with increased mortality, adverse effects, and emergence of drug resistant isolates (11, 12). However, tigecycline is still reserved as a last-resort drug in the treatment of severe and complicated infections caused by *A. baumannii* (12, 13). Data regarding the clinical use of tigecycline in the treatment of *Acinetobacter* species are scarce and are confounded by the use of the drug in combination regimens. Also, the potential development of resistance to tigecycline during the course of therapy is of a concern (14). Although tigecycline has shown considerable, though not consistent, antimicrobial activity against multidrug resistant (MDR, including carbapenem resistant) *Acinetobacter* species, the ultimate role of tigecycline in the treatment of MDR *Acinetobacter* species remains undefined (13–15). Therefore, well designed studies on the clinical use of tigecycline in the treatment of infections caused by MDR *Acinetobacter* species are warranted (14).

Polymyxins such as colistin can be also used as last-resort drugs in severe infections caused by *A. baumannii* (16). However, studies have shown that colistin monotherapy is unable to prevent resistance and does not influence the 30-day mortality in patients with MDR *A. baumannii* (MDRAB) bloodstream infections (17, 18). Therefore, combination therapy such as colistin and rifampicin or colistin and carbapenem may be the best antimicrobial strategy against colistin resistant *A. baumannii* infections (17).

### Prevention of *A. baumannii* infections

Decreasing the incidence of *A. baumannii* infections can be achieved by applying the following preventive measures: (1) strict infection control and contact precaution policies, (2) appropriate use of invasive procedures, and (3) appropriate utilization of antimicrobial agents (2, 3, 5, 8). Spread of MDRAB can be limited by: (1) enforcement of aggressive infection control measures, (2) development of innovative control strategies, (3) education of staff, patients, and visitors, (4) hand washing and use of antiseptics, (5) surveillance cultures from patients, staff, and environment, and (6) cleaning and disinfection of hospital equipment (2, 3, 5, 8). Eradication of *Acinetobacter* species requires adherence to good infection control practices, prudent antibiotic utilization, and effective antimicrobial therapy (2, 3, 5, 8).

### Prognosis of *A. baumannii* infections

The prognosis of *A. baumannii* infections varies considerably. Good prognostic factors include: local infection or trauma, absence of MDR strains, medical conditions other than malignancy or burns, and prompt as well as efficient antibiotic therapy (3). Poor prognosis is associated with: age >65 years, underlying medical condition being malignancy or burns, presence of virulent or MDR strain, late or inappropriate antimicrobial therapy, DIC or coagulopathy, bacteremia or septic shock, mechanical ventilation, and rapidly progressive or ultimately fatal illness (3). In patients having *A. baumannii* infections, high mortality rates are associated with: pneumonia, bacteremia, and inappropriate medical treatment. The ultimate outcome of *A. baumannii* infections correlates well with: the type of underlying medical illness, presence or absence of polymicrobial bacteremia, and appropriate or inappropriate antimicrobial therapy (3).

## Drug Resistance Exhibited by *A. baumannii*

### Emergence of drug resistance

*Acinetobacter baumannii* is characterized by frequent MDR due to multiple mechanisms. Isolates of *A. baumannii* have shown high levels of antimicrobial resistance, particularly to β*-*lactam agents (3, 19). Antimicrobial resistance among *Acinetobacter* species has substantially increased in the last decade. In a European survey, resistance of *A. baumannii* rated number 5 among the evolving bacterial resistance to antimicrobial therapy (3, 19). The patterns of antimicrobial resistance exhibited by *A. baumannii* isolates vary among distinct geographical regions, especially for nosocomial isolates. Approximately 11% of nosocomial isolates of *A. baumannii* are resistant to carbapenems (20).

### Genetic resistance

With the advent of whole-genome sequencing, important gains in the insights of genetic complexity of *A. baumannii* have been obtained (1). Its wide array of drug resistance determinants and its ability to effectively regulate these according to selective environmental pressures clearly demand respect. The global epidemiology of *A. baumannii* is of a concern for widespread dissemination, most often in a clonal manner within institutions, cities, and sometimes between countries (1). In patients with bacteremia caused by *A. baumannii*, complex genotype 2 is associated with greater resistance and higher mortality compared to other genospecies, particularly genospecies 13 TU. In critically ill patients with *A. baumannii* bacteremia, genospecies 2 is significantly associated with pneumonia while genotype 13 TU is associated with primary bacteremia (21).

### Mechanisms of drug resistance

*Acinetobacter baumannii* has various mechanisms of resistance for different classes of antimicrobials. Examples of mechanisms of drug resistance to various classes of antibiotics are shown in Table 2 (1, 22–27). MDR determinants as well as genetic determinants of drug resistance for *A. baumannii* are shown in Table 3 (22, 24–27).

**Table 2:** **Mechanisms of *A. baumannii* drug resistance**.

Class of antibiotic	Mechanisms of resistance
(1) β-Lactams	β-Lactamases
	Outer membrane proteins
	Efflux pumps
	Altered penicillin-binding proteins
(2) Aminoglycosides	Aminoglycoside modifying enzymes
	Efflux pumps
	Ribosomal [16S rRNA] methylation
(3) Quinolones	Efflux pumps
	Modification to target binding site
	Genetic mutations such as gyrA and parC
(4) Tetracyclines and glycylcyclines	Multidrug efflux pumps
	Tetracycline-specific efflux
	Ribosomal protection

**Table 3:** **Determinants of drug resistance in *A. baumannii***.

Type of determinant	Examples
1. Multidrug resistance determinants	Weak permeability
	Efflux systems
	Enzymatic mechanisms that comprise production of:
	(A) β-Lactamases such as OXA-23 carbapenemase
	(B) Arm A 16S rRNA methylase
2. Genetic determinants of resistance	Genetic mutations such as gyrA and parC
	Insertion sequences
	Novel genetic elements such as chromosomal resistance genetic islands

### MDR *A. baumannii*

*Acinetobacter* species possess a wide array of β*-*lactamases that hydrolyze and confer resistance to penicillins, cephalosporins, and carbapenems (3). MDR is defined as non-susceptibility to at least three antimicrobials; such as ampicillin–sulbactam, piperacillin–tazobactam, ceftazidime, imipenem, meropenem, aminoglycosides, ciprofloxacin, aztreonam, and trimethoprim–sulfamethoxazole; which are routinely tested in the clinical laboratory and to which *A. baumannii* would have been expected to be susceptible (22, 28, 29). Risk factors for evolution of MDRAB infections include: (1) previous use of carbapenems and third generation cephalosporins, (2) recent insertion of a CVC, (3) endotracheal intubation and mechanical ventilation, (4) recovery of *A. baumannii* from various sites, and (5) bacteremia caused by other microorganisms (3, 28, 29). MDRAB infections carry a high crude mortality rate and have a great impact on health care settings as they are most frequently encountered in severely ill patients (3). Several outbreaks of MDRAB have been reported in USA. The Infectious Diseases Society of America has recently identified *A. baumannii* as one of the six particularly problematic pathogens in terms of antimicrobial susceptibility issues arising from resistance (22). Sequence type 92 (ST92) and the associated clonal complex 92 represent the most sampled and widespread sequence types and are known as European clone 2 and worldwide clonal lineage 2. Three clonal complexes were initially described in Europe then documented in North America, Asia, Africa, and Australia (30). The coexistence of several resistance determinants may also present a significant threat (24).

The potentially effective antimicrobial agents against MDRAB infections include: carbapenems; aminoglycosides such as gentamicin and amikacin; tetracyclines such as doxycycline and minocycline; sulbactam; colistin; and tigecyclines. However, drug combinations are preferable and, in particular, the combination of carbapenems and colistin is becoming the treatment of choice (3, 6, 22). Therapeutic options for MDRAB infections are limited and there are no controlled trials to guide the therapeutic choice. Therefore, well designed clinical studies are necessary to guide clinicians on decisions regarding the best therapeutic option for patients with MDRAB infections (3, 6, 22).

Ciclopirox, a topical antifungal agent that was developed 40 years ago, has multiple potential uses including treatment of human immunodeficiency virus (HIV), enhancement of wound healing in diabetics, and potential use in the treatment of multiple myeloma. It has been found to be effective in the treatment of MDRAB infections (31). Recently, a number of studies have focused on non-antibiotic approaches that utilize novel mechanisms of action to achieve antibacterial activity against MDR bacteria (32). Modern advances in phage therapy, iron chelation treatment, antimicrobial peptides, prophylactic vaccination, photodynamic therapy, and nitric oxide-based treatments have also shown promising activity against MDRAB (32). The siderophore sulbactam [BAL 30072] has shown promising *in vitro* activity against MDRAB isolates harboring AmpC and OXA β*-*lactamases (33).

### Carbapenem-resistant *A. baumannii*

The emergence of carbapenem-resistant *A. baumannii* (CRAB) was first reported in USA in 1991 (34). CRAB has been linked to point multations in porin channels from the outer membrance. These point mutations alter bacterial targets of functions thus decreasing the affinity for antimicrobial agents or upregulating cellular functions by producing efflux pumps or other proteins (3). Spread of CRAB in Asia has been linked to global clone 2 (GC2) and Aba R-4 type resistance islands (35, 36). CRAB has also been attributed to the expression of β-lactamases; such as OXA-23, OXA-24/40, and OXA-58; which inactivate carbapenems (37). Outbreaks of CRAB infections in hospital settings illustrate the important role of post-acute care facilities in dissemination of MDR bacterial pathogens (37).

Although imipenem is the most active agent against *A. baumannii*, resistance to imipenem is becoming increasingly common and hospital outbreaks of imipenem resistant MDRAB (IR-MDRAB) have been increasingly reported since the early 1990s. Risk factors for the development of IR-MDRAB infection include: previous ICU admission, exposure to third generation cephalosporins, and use of carbapenems (3). The time at risk, the period of time at risk for appearance of IR-MDRAB, is a very important confounding factor to be adjusted because the probability of appearance increases with the length of time (3). The best therapeutic approach for CRAB infection is to use colistin in combination with tigecycline or cefoperazone–sulbactam or piperacillin–tazobactam (38).

### Pandrug resistance of *A. baumannii*

Pandrug resistant *A. baumannii* (PDRAB) has emerged as an important cause of both endemic HCAIs as well as epidemic outbreaks (39). PDRAB infections are associated with high morbidity and mortality. In addition, their treatment has high costs due to the utilization of ICU facilities and potent antimicrobial therapies (39). Isolates of PDRAB were first reported in 1998 in Taiwan (34). PDRAB strains are usually resistant to all antibiotics that are routinely used including: ampicillin–sulbactam; ceftazidime and cefepime; piperacillin–tazobactam; aztreonam; fluoroquinolones including ciprofloxacin, travofloxacin, and moxifloxacin; garenoxacin as well as amikacin and carbapenems including imipenem and meropenem (34, 40–42). The increased utilization of carbapenems and fluoroquinolones as well as clonal dissemination may explain the recent spread of PDRAB strains (40–42).

The British Society of Antimicrobial Chemotherapy has provided criteria to designate MICs for tigecycline-susceptible-and-resistant *A. baumannii* isolates as ≤1 and >2 μg/ml, respectively. Implementation of these breakpoints would limit the use of tigecycline to salvage therapy for certain infections caused by PDRAB (16). Novel therapies for PDRAB or extensively drug resistant *A. baumannii* (XDRAB) infections include: (1) drug combinations containing colistin or polymyxin B, which are composed of two or three drugs including imipenem, rifampicin, amikacin, or ampicillin–sulbactam in addition to colistin or polymyxin B and (2) drug combinations that are composed of tigecycline in addition to two other drugs including: imipenem, amikacin, and cefepime (43, 44). Aggressive early treatment of PDRAB with adequate doses of drugs in combinations such as carbapenem–sulbactam may prevent the emergence of PDRAB strains (45). A multifaceted approach or intervention that includes active surveillance, environmental cleaning, and appropriate utilization of antimicrobials appears to be efficient and cost–effective in the management of PDRAB infections (39).

## *Acinetobacter baumannii* Bacteremia

### Sources of bacteremia and epidemiology

*Acinetobacter baumannii* bacteremia may be primary, where no cause is found, or secondary to infections involving wounds, respiratory, and urinary tracts in addition to other sites of infection (1, 3). *A. baumannii* bloodstream infections are more common in ICU settings than in general hospital wards and are more frequent in hospitals than in the community. They usually develop at a mean of 26 days from the time of hospital admission (1). In a large study of nosocomial blood stream infections in the USA (1995–2002), *A. baumannii* ranked the 10th most common etiological agent being responsible for 1.3% of all monomicrobial bloodstream HCAIs (1). However, only 62% of *A. baumannii* bacteremias in hospitalized patients are considered clinically significant. Therefore, it is essential to perform thorough clinical evaluation of patients to eliminate the possibility of pseudobacteremia (3). Additionally, approximately 30% of patients with *A. baumannii* bacteremia have polymicrobial bacteremia or other infections (3).

### Risk factors for *A. baumannii* bacteremia

There are several risk factors for the development of *A. baumannii* bacteremia and these are shown in Table 4 (3, 29, 46). The risk factors for mortality related to *A. baumannii* bacteremia are variable and they include: (1) advanced age, (2) recent surgery,(3) immunosuppressive status, (4) invasive procedures such as CVC, urinary catheterization, pulmonary catheterization, mechanical ventilation, and nasogastric tubes, (5) presence of complications such as septic shock, DIC, acute renal failure, and acute respiratory failure, (6) use of inappropriate antibiotic therapy, (7) genotype 2, (8) low platelet count, (9) low serum albumin concentration, (10) the number of comorbid medical conditions, and (11) high acute physiology and chronic health (APACHE) Π and Hilf’s severity scores on admission to ICU (21, 47–50).

**Table 4:** **Risk factors for *A. baumannii* bacteremia**.

(1) Immunosuppression
(2) Unscheduled admission to hospital
(3) Prior antimicrobial therapy
(4) Previous ICU sepsis
(5) Development of septicemia or septic shock
(6) Respiratory failure on admission to ICU
(7) Use of invasive procedures
(8) Endotracheal intubation and mechanical ventilation
(9) Recent insertion of a CVC
(10) Bacteremia caused by other microorganisms after colonization by MDRAB

*CVC, central venous catheter; MDRAB, multidrug resistant *Acinetobacter baumannii**.

### Clinical course in patients with *A. baumannii* bacteremia

In patients with *A. baumannii* bacteremia, the overall mortality rate ranges between 25 and 54% and mortality depends on a number of factors that include general clinical condition of the patient, whether the patient is managed in general wards or in ICU and whether the organism is susceptible or resistant to antimicrobial therapy (1, 3, 48, 49, 51, 52). Studies have shown that crude mortality rate ranges between 5 and 16.3% outside ICU while in ICU, it ranges from 34 to 70% and may even reach 91.7% in case the organism is MDR. However, in critically ill patients, attributable mortality to *A. baumannii* bacteremia ranges between 7.8 and 19% (1, 3, 48, 49, 51, 52).

### MDRAB bacteremia

Risk factors for MDRAB bacteremia include: prior ICU admission and prior use of broad-spectrum antibiotics, particularly carbapenems, β-lactams, and inhibitors of β-lactamases. Independent risk factors for mortality in patients having MDRAB bacteremia include high APACHE Π score and presence of secondary rather than primary bacteremia (52). Septic shock has been reported in 42% of patients having *A. baumannii* bloodstream infections. Complicated clinical course and life-threatening complications are more likely to evolve in immunocompromised individuals (3). In patients with *A. baumannii* bacteremia, good prognosis is associated with infections related to CVCs where survival may reach 96.2% while poor outcome is associated with inappropriate utilization of antimicrobial therapy and presence of respiratory tract infections where mortality rate may reach 39.2% (48, 50). Sources of *A. baumannii* bacteremia in ICU settings include primary bacteremia, respiratory tract, wounds, and intravascular catheters (47). Risk factors for *A. baumannii* in ICU patients include invasive procedures, use of broad-spectrum antimicrobials, and the critical condition of the patient i.e., immunosuppression and comorbid medical conditions. In ICU patients, *A. baumannii* bacteremia carries a high mortality rate and although imipenem is active against *A. baumannii* infections, the increasing resistance to carbapenems, particularly in ICU setting, is alarming (47).

Sequential organ failure assessment (SOFA) and APACHE scores determined at the onset of *A. baumannii* bacteremia are reliable risk stratification tools that predict 14-day and in-hospital mortality (3). The invasive procedure index, the number of invasive procedures performed everyday during the ICU stay before the onset of *A. baumannii* bacteremia divided by the number of days spent in the ICU before the onset of *A. baumannii* bacteremia, is also important in determining the outcome of patients having bacteremia (46). Early identification of patients at high risk of mortality based on a variety of risk factors is important. Consequently, treatment strategies should be based on risk stratification of patients having *A. baumannii* bacteremia (50).

### Treatment of *A. baumannii* bacteremia

The treatment of choice for *A. baumannii* bacteremia is not yet established. In patients having infected CVCs, removal of these catheters is indicated (48). Concerns have been raised about the use of tigecycline is treating *A. baumannii* bloodstream infections thus leaving colistin as the only therapeutic option for some of these infections (1).

### *Acinetobacter baumannii* bacteremia in HSCT recipients

In recipients of HSCT, the risk factors for the development of *A. baumannii* bacteremia include: (1) severe underlying illness such as hematologic malignancy, (2) immunosuppressive therapies such as corticosteroids and cyclosporine-A, (3) graft versus host disease (GVHD), (4) colonization of respiratory, gastrointestinal, and urinary tracts, (5) prolonged use of broad-spectrum antibiotics, and (6) invasive instrumentation such as CVCs and endotracheal intubation (3, 53). In a single center retrospective study that included 483 HSCT recipients and performed over 6 years, 19 patients developed MDRAB bacteremia after engraftment, pneumonia was the origin of bacteremia in all patients and 95% of patients with bacteremia and 8.3% of patients without bacteremia died. The risk factors for the development of MDRAB bacteremia in HSCT recipients were history of care in ICU after HSCT and the time duration between admission to hospital and HSCT (53).

## Novel Therapies for *A. baumannii* Infections

The global emergence of MDRAB necessitates the development of novel preventive and therapeutic strategies to control infections caused by this pathogen. Recently, there has been increased interest in non-antibiotic approaches that utilize novel mechanisms of action to achieve antibacterial activity (54). Recent advances in the following potential therapeutic modalities have shown activity against *A. baumannii*: (1) phage therapy, (2) iron chelation treatment, (3) antimicrobial peptides, (4) prophylactic vaccination, (5) photodynamic peptides, (6) decontamination with hydrogen peroxide, and (7) nitrous oxide-based therapies (54–57). However, these therapeutic approaches have their own limitations and long-term safety must be addressed before utilization of these novel therapies in the clinical arena (54).

Phages have been used as pharmaceutical agents for more than 90 years. Evolution of MDR bacterial infections has renewed interest in the utilization of living phages in the treatment of infectious diseases in humans and animals (55). The development of a phage cocktails therapy, such as phage Φm18p, could be an alternative therapeutic modality to antibiotics in the management of life-threatening bacterial infections in the future (55). The novel lipoglycopeptide [telavancin] has shown remarkable synergistic activity once combined with polymyxins, such as colistin, in the treatment of GNB infections including those caused by MDRAB (56). Therefore, glycopeptide–polymyxin combinations may become a useful therapeutic option in the treatment of infections caused by MDR–GNB (56).

Currently, colistin and tigecycline are the drugs of choice for MDRAB infections. However, these drugs have their own limitations, side effects in addition to the potential of development of drug resistance (55, 58). Colistin and intravenous colistimethate sodium are peptide antibiotics that can be used as a last-resort treatment of infections caused by MDRAB (58).

Comprehensive infection control measures, combined with environmental decontamination using vaporized hydrogen peroxide, can interrupt the cycle of transmission of MDRAB within long-term acute health care facilities (57). Silver carbene complexes and their nanoparticles have shown activity against clinical isolates of several MDR bacteria including *A. baumannii* (59). Their low toxicity and increased antimicrobial activity against a multitude of virulent pathogens facilitates their future application as potential targeted therapies for serious infections caused by MDR bacteria (59).

AntibacTR is a computation pipeline composed of a database and web-based tool for ranking of proteins present in GNB. It aids researcher working in the field of antibacterial drug discovery to select potential drug targets (60). It is versatile and integrates both experimental annovation and computational analysis. Currently, the database covers 74 GNB including *A. baumannii* (60).

## Donor Granulocyte Transfusion Therapy

Antibiotic refractory bacterial infections and opportunistic fungal infections are important causes of morbidity and mortality in neutropenic patients (61). Polymorphonuclear leukocytes (DMNLs) play a vital role in host defense against various infectious agents (61). The relationship between the degree and duration of neutropenia and the risk of infections had been observed since the 1960s (62). However, the efficacy and feasibility of donor granulocyte transfusion therapy (GTX) has changed considerably over the past five decades (63). Since the late 1990s, there has been renewed interest due to the increasing demand for GTX following several reports of efficacy of this procedure to treat and prevent severe infections in patients with neutropenia (64, 65). Despite the lack of solid evidence, GTXs are consistently used as adjunctive therapy of severe, persistent, and progressive infections in neutropenic individuals (62, 65, 66). Response rates ranging between 30 and 83% have been observed in neutropenic patients with severe and uncontrolled infections but overall survival was mostly determined by the underlying disease process and the time of endogenous neutrophil recovery (62, 66). Bacterial infections consistently responded better than fungal infections and patients having GN organisms responded better than those having Gram-positive bacterial infections (62, 65). GTXs and granulocyte stimulating factors have successfully been used as adjunctive therapies in patients with severe aplastic anemia (SAA) having serious bacterial and fungal infections (67–69).

Despite the presence of multiple predictors of increased mortality in cancer patients with candidemia, high-dose GTXs in these high-risk patients has been associated with better than expected survival rates (63). GTXs have also been used prophylactically in neutropenic patients despite the absence of solid evidence to support pre-emptive utilization of donor GTXs (62, 70). However, results of the few published studies reported positive outcome but no significant difference with respect to duration of hospitalization or 100-day survival has been reported (62, 70).

Despite numerous clinical trials on the use of GTXs in treating bacterial and fungal infections in neutropenic patients, the safety and efficacy of this potential therapeutic modality remain controversial (61, 71). Therefore, there is a need for controlled prospective trials to evaluate the best time to give donor GTXs, the role of donor granulocytes as pre-emptive-empiric therapy and the optimal duration of adjunctive high-dose GTX in selected high-risk patients having systemic infections (63).

The following minimal criteria are usually used to justify the use of donor GTXs: (1) absolute neutrophil count 500 × 10^9^/L except in case of chronic granulomatous disease, (2) evidence of bacterial or fungal infection: clinical symptoms of infection, positive cultures, pathological diagnosis of infection made by biopsies taken from involved sites, and radiological evidence of infection such pneumonia, and (3) unresponsiveness to antimicrobial therapy for at least 48 h except in extreme circumstances with life-threatening infections (72, 73).

Two major non-randomized trials on the use of donor GTXs have been published. The first one, phase I/II trial in neutropenic patients with severe infections included 30 patients, whose infections had not been controlled with adequate antibiotics and G-CSF administration. Following granulocyte transfusions: bacterial and fungal infections were controlled in 82 and 38% of patients, respectively and 100-day survival in patients having bacterial and fungal infections were 82 and 54%, respectively (72, 74). The second one, phase I/II non-randomized trial, showed that resolution of infection occurred in 8 of 11 patients with invasive bacterial and fungal infections and that 50% of patients who received GTXs following HSCT survived until engraftment. However, none of the five patients with invasive aspergillosis cleared the infection, but no evidence of infection was found at autopsy (72, 75).

A Cochrane meta-analysis of eight randomized clinical trials on the use of donor GTXs in neutropenic patients concluded that the available evidence was insufficient to either support or refute the generalized use of GTXs in the most common neutropenic patient populations i.e., patients receiving myeloablative chemotherapy and/or HSCT (72, 76).

The national heart, lung, and blood institute (NHLBI) has recently sponsored a large multicenter, phase III randomized clinical trial (the RING study: resolving infections in people with neutropenia using high-dose granulocyte transfusions), with the aim to evaluate the incremental benefits of donor GTXs in patients having bacterial and fungal infections post-HSCT (62, 67, 72). The study has already finished accrual and the results are awaited. The results of this clinical trial are expected to have a great impact on the use of GTXs in neutropenic patients having severe fungal and bacterial infections (62, 67, 72).

Until the results of prospective randomized controlled trials including the RING study are available, the use of donor GTXs in patients with bone marrow failure, hematological malignancies and in recipients of HSCT having septic neutropenia or serious bacterial and fungal infections will continue (67, 72). However, GTXs should be given in specific institutions according to well established and preferably standardized operational procedures in order to ensure safety of both donors and recipients (62).

Complications of GTXs in recipients include: (1) allergic reactions such as fever and hypotension, (2) pulmonary complications such as respiratory distress, pulmonary infiltrates, pulmonary edema, and transfusion-related acute lung injury, (3) transfusion-related GVHD due to presence of donor lymphocytes in granulocyte concentrates, (4) alloimmunization due to formation of HLA antibodies directed against granulocyte-specific antigen, and (5) transmission of infectious agents such as cytomegalovirus (CMV) which is harbored in peripheral blood leukocytes (61, 62, 65, 66, 72).

In recipient of HSCT having donor GTXs, the main concerns are: (1) GVHD which is a potential complication of granulocyte transfusion, (2) alloimmunization and formation of HLA antibodies directed against granulocyte-specific antigen (61, 66, 72), and (3) the effect of transfusion-associated leukocyte compatibility on the clinical outcome of HSCT (61). Patients, who had previously received GTX from incompatible donors, experienced delayed PMNL engraftment and more febrile episodes following HSCT (61). In neutropenic HSCT recipients, serial granulocyte transfusions have been successfully used in the treatment of MDR bacterial infections causing septic complications (77). In recipients of HSCT having invasive fungal infections, GTXs have also been successfully used in combination with antifungal agents to control these serious infections (78, 79).

Allogeneic HSCT recipients have defects involving different components of their immune system which subsequently increase the risk of having invasive fungal infections (80). Neutropenia is the single most important risk factor for the development of invasive fungal infections in recipients of HSCT (80). One of the potential approaches that aid in restoration of immunity and help in fighting fungal infections is the administration of granulocytes that have donor-derived antifungal T-cells (80, 81).

## Conclusion

*Acinetobacter baumannii* has recently emerged as a major cause of morbidity and mortality in hospitalized immunocompromised individuals. Increasingly reported drug resistance exhibited by the organism is gaining global dimensions and complicates the management of infections caused by this GNB. Patients with hematological malignancy and recipients of various types of HSCT are at high risk for development of infectious complications related to *A. baumannii*. Appropriate utilization of antimicrobials, provision of advanced supportive care, and application of strict infection control measures are essential in the management of infections caused by *A. baumannii*.

## Conflict of Interest Statement

The authors declare that the research was conducted in the absence of any commercial or financial relationships that could be construed as a potential conflict of interest.
